# *PLOS Pathogens* 2018 Reviewer and Editorial Board Thank You

**DOI:** 10.1371/journal.ppat.1007649

**Published:** 2019-02-27

**Fig 1:**
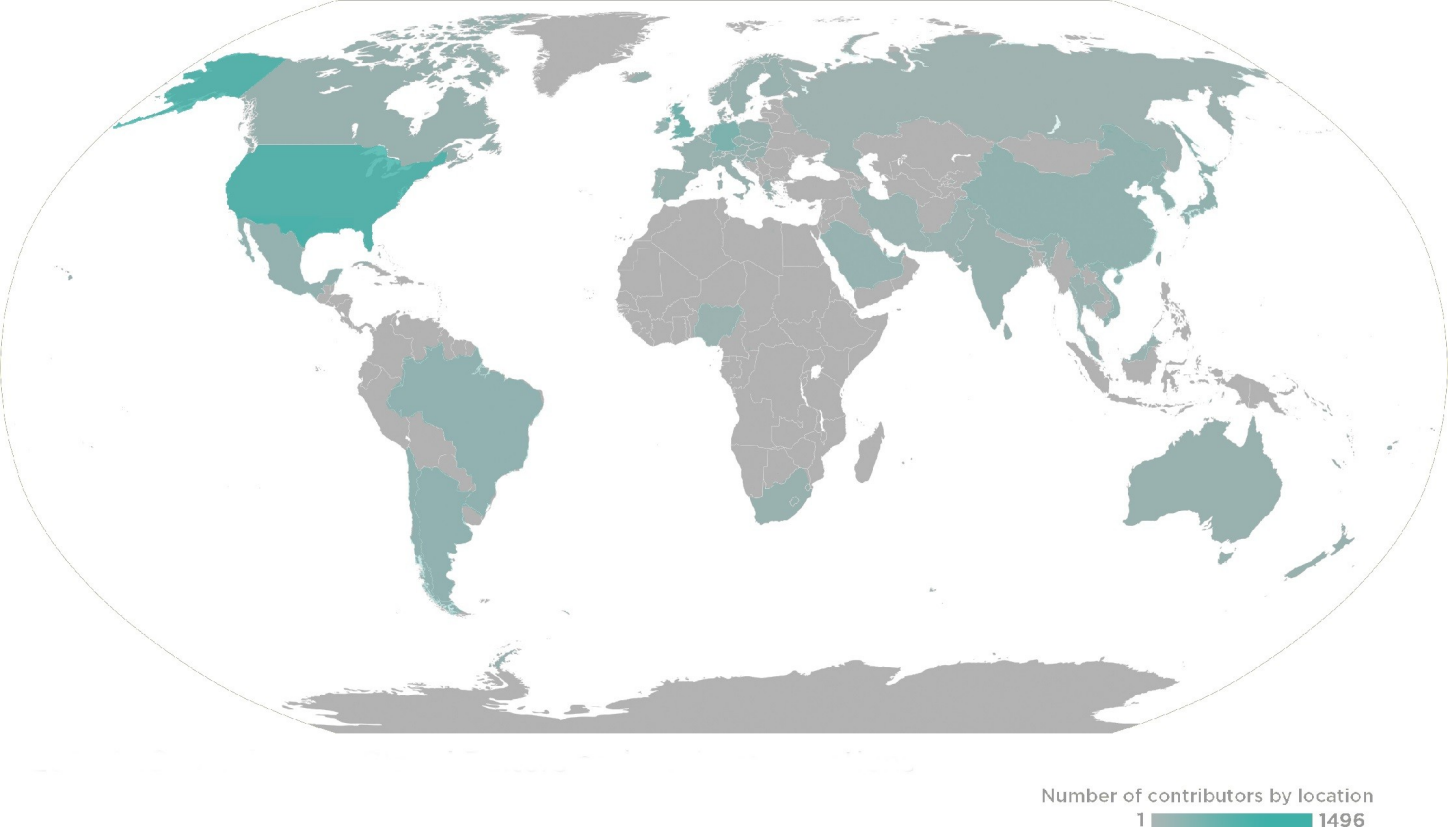
2018 *PLOS Pathogens* Global Editor and Reviewer locations.

PLOS and the *PLOS Pathogens* team want to sincerely thank all of our Editorial Board Members, Guest Editors, and Reviewers for the journal in 2018. Your contributions of time and expertise support your research community, advance scientific progress, and continue to make *PLOS Pathogens* a leader in its field. This past year, *PLOS Pathogens* received the assistance of 233 Editorial Board members, 264 Guest Editors, and 2,064 Reviewers, who handled 2,256 manuscripts that resulted in 579 publications (Fig 1).

We’re deeply grateful to all of our volunteers whose dedicated efforts support *PLOS Pathogens* and Open Science. Thank you all for your work!
